# A randomized controlled trial of in-patient treatment for anorexia nervosa in medically unstable adolescents

**DOI:** 10.1017/S0033291714001573

**Published:** 2014-07-14

**Authors:** S. Madden, J. Miskovic-Wheatley, A. Wallis, M. Kohn, J. Lock, D. Le Grange, B. Jo, S. Clarke, P. Rhodes, P. Hay, S. Touyz

**Affiliations:** 1 Eating Disorder Service at The Sydney Children's Hospitals Network, Westmead, Australia; 2 Discipline of Pediatrics, Faculty of Medicine, The University of Sydney, Australia; 3 Westmead Clinical School, The University of Sydney, Australia; 4 Centre for Research into Adolescents’ Health (CRASH), Adolescent Medicine Unit, Westmead Hospital, Australia; 5 Psychiatry and Behavioral Science, School of Medicine, Stanford University, USA; 6 Department of Psychiatry and Behavioral Neuroscience, The University of Chicago, USA; 7 School of Psychology, The University of Sydney, Australia; 8 Centre for Health Research, School of Medicine, The University of Western Sydney and School of Medicine, James Cook University, Australia

**Keywords:** Anorexia nervosa, family-based treatment, in-patient treatment, medical instability, treatment optimization

## Abstract

**Background:**

Anorexia nervosa (AN) is a serious disorder incurring high costs due to hospitalization. International treatments vary, with prolonged hospitalizations in Europe and shorter hospitalizations in the USA. Uncontrolled studies suggest that longer initial hospitalizations that normalize weight produce better outcomes and fewer admissions than shorter hospitalizations with lower discharge weights. This study aimed to compare the effectiveness of hospitalization for weight restoration (WR) to medical stabilization (MS) in adolescent AN.

**Method:**

We performed a randomized controlled trial (RCT) with 82 adolescents, aged 12–18 years, with a DSM-IV diagnosis of AN and medical instability, admitted to two pediatric units in Australia. Participants were randomized to shorter hospitalization for MS or longer hospitalization for WR to 90% expected body weight (EBW) for gender, age and height, both followed by 20 sessions of out-patient, manualized family-based treatment (FBT).

**Results:**

The primary outcome was the number of hospital days, following initial admission, at the 12-month follow-up. Secondary outcomes were the total number of hospital days used up to 12 months and full remission, defined as healthy weight (>95% EBW) and a global Eating Disorder Examination (EDE) score within 1 standard deviation (s.d.) of published means. There was no significant difference between groups in hospital days following initial admission. There were significantly more total hospital days used and post-protocol FBT sessions in the WR group. There were no moderators of primary outcome but participants with higher eating psychopathology and compulsive features reported better clinical outcomes in the MS group.

**Conclusions:**

Outcomes are similar with hospitalizations for MS or WR when combined with FBT. Cost savings would result from combining shorter hospitalization with FBT.

## Introduction

Anorexia nervosa (AN) has a lifetime prevalence of 0.9–2.3% with its onset primarily in adolescence (Lewinsohn *et al.* 2000; Wade *et al.* 2006; Keski-Rahkonen *et al.* 2007; Preti *et al.* 2009). AN is the third most common chronic disorder affecting adolescent females, with an average mortality rate of 5% (Steinhausen, 2002). Treatment costs are among the highest of all psychiatric disorders due to the extensive use of hospitalization (Striegel-Moore *et al.* 2000; Agras, 2001; Crow & Nyman, 2004). Although hospitalization for the management of acute medical instability (e.g. hypothermia, hypotension, bradycardia, electrolyte abnormalities and cardiac arrhythmias) may be essential in preventing morbidity and mortality (Golden *et al.* 2003; Katzman, 2005), the benefits of further hospitalization for weight normalization are unclear.

There are few studies on the role of hospitalization in AN and no randomized controlled trials (RCTs) comparing different in-patient interventions. Results from uncontrolled studies in adolescents are contradictory, with some suggesting that hospitalization to near normal weight improves outcomes and decreases the need for hospitalization over the course of the illness (Steinhausen *et al.* 2008), and others suggesting that hospitalization is not better than out-patient treatment (Crisp *et al.* 1991; Gowers *et al.* 2007) or is associated with poorer outcomes (Gowers *et al.* 2000). Given this lack of evidence, it is therefore not surprising that hospitalization practices vary considerably around the world, with length of stay based on local expert consensus and economic imperatives including treatment costs and insurance coverage. Reported lengths of stay in European studies are considerably longer than in the USA, particularly in adolescents. A 25-year review of eating disorder admissions in Iceland reported an average length of hospital stay of 67.3 days in AN adults and 129.7 days in AN adolescents (Sigurdardottir *et al.* 2010). Similarly, in France, a retrospective review of a large, specialist child and adolescent eating disorder unit reported a mean length of stay of 135 days (Stirk Lievers *et al.* 2009), whereas in a multi-center RCT of adolescent AN treatment in the UK, the average length of hospitalization was 106.4 days (Gowers *et al.* 2010). In the USA, where hospital admission for AN is often limited to medically unstable patients, lengths of stay tend to be brief. Thus, a review by Chu *et al.* (2012) of admissions in medically unstable adolescents and adults in the USA reported an average length of stay of 16 days. However, as a result of shorter hospitalization, there is a growing trend for patients in the USA to move to non-hospital-based residential treatment settings where the average length of stay has been reported to be 83 days (Frisch *et al.* 2006).

Because of the high financial costs and potentially negative effects of either too much or too little hospitalization on treatment outcomes, in particular the detrimental impact of prolonged hospitalization on adolescent development, it is imperative to understand the impact of different lengths of hospitalization on outcomes in adolescent AN. In a RCT comparing in-patient and out-patient treatments for adolescent AN, Gowers *et al.* (2010) identified the question of optimum length of hospitalization in this group as one of the key research issues for this group of patients.

Family-based treatment (FBT) is an evidence-based out-patient treatment for adolescent AN with demonstrated efficacy in six RCTs with a 2–5-year follow-up, in adolescents aged 12–19 years with less than a 3-year history of AN (Russell *et al.* 1987; Le Grange *et al.* 1992, 2010; Eisler *et al.* 1997, 2000, 2007; Lock *et al.* 2005, 2010; Le Grange & Eisler, 2009). These studies suggest that FBT is effective in both maintaining weight and improving eating disorder psychopathology following hospital discharge in weight-restored adolescents and in treating medically stable but underweight adolescents with AN as out-patients, including patients at low weights. Furthermore, FBT is reported to reduce hospital readmission rates (Wallis *et al.* 2007; Lock *et al.* 2008).

The aim of this study was to compare the effectiveness of different in-patient treatments in medically unstable adolescents with AN prior to out-patient FBT by conducting an RCT comparing brief hospitalization for medical stabilization (MS) to hospitalization for weight restoration (WR) to 90% expected body weight (EBW) for gender, age and height, both followed by FBT. Our primary outcome was the number of hospital days following initial hospitalization at the 12-month follow-up. We hypothesized that those randomized to WR would require fewer total hospital days post-discharge, fewer readmissions and fewer total hospital days over the course of treatment than those randomized to MS following their initial hospitalization.

## Method

This two-site study [the Sydney Children's Hospitals Network, Westmead Campus (SCHN-W) and Westmead Hospital (WH)] randomized 82 participants to either hospitalization for MS or hospitalization for WR to 90% EBW (1:1), both followed by FBT. Following consent (assent for adolescents aged < 18 years), participants were randomized in clusters of six using a block size of two. Each new cluster was randomized through a blind random binary list created by an external statistician. Although the use of clusters unblinded recruitment staff to the group status of participants, this design was chosen to prevent potential problems of drop-out if participants from different groups were treated alongside one another in hospital and became dissatisfied with their allocation. Patients and families were blind to treatment assignment prior to randomization. A clinical psychologist blind to treatment assignment conducted all baseline interviews and collected questionnaires at assessment time points.

### Participants

Participants were recruited from 266 consecutive eating disorder admissions to two specialist pediatric medical units between June 2007 and February 2010. Participants were eligible if they were aged between 12 and 18 years, with a DSM-IV diagnosis of AN of less than 3 years’ duration (APA, 2000), were medically unstable [hypothermic (temperature < 35.5°C), bradycardic (heart rate < 50 beats/min), hypotensive (blood pressure < 80 mmHg systolic and < 40 mmHg diastolic), orthostatic instability (pulse increase > 20 beats/min, systolic blood pressure decrease > 20 mmHg) or QT interval corrected for heart rate > 0.45 s] (Baran *et al.* 1995), lived within a 2-h drive of the treatment center to allow for weekly participation in FBT and were not receiving other psychotherapy during the RCT. All participants would have met DSM-V criteria for AN (APA, 2013). Exclusion criteria included an illness duration of more than 3 years, evidence of psychosis, mania, substance abuse or significant intercurrent medical illnesses other than nutrition-related complications of AN. No patients were excluded because of intercurrent medical illnesses or co-morbid psychiatric conditions.

**Fig. 1.**
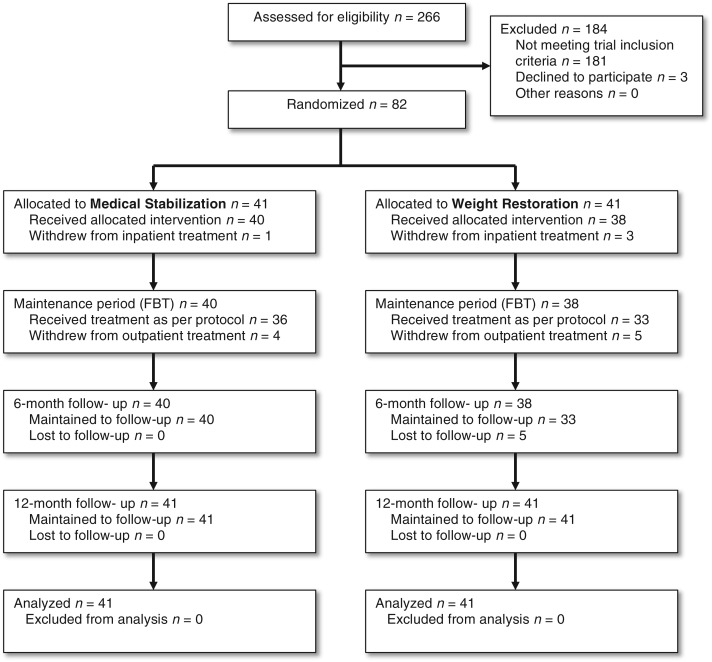
Consortium diagram of patient flow.

Weight thresholds (<85% EBW) for study entry were calculated using the Centers for Disease Control and Prevention (CDC) growth charts for expected weight for gender, age and height (Kuczmarski *et al.* 2000). The study was described in detail to eligible participants (85) and their families and 82 (96%) were randomized (see Fig. 1). All patients and their families were made aware that they would continue to receive in-patient hospitalization and the opportunity to receive FBT if they did not participate in the trial.

### Assessments and procedures

Assessment included diagnostic evaluation for eating disorder symptomatology and co-morbid psychiatric disorders, medical assessment and standardized psychological questionnaires. There were five assessment points: at hospital admission, hospital discharge, end of FBT (session 20), 6- and 12-month follow-ups. Medical assessments were conducted by pediatricians experienced in the management of eating disorders and psychiatric assessments by an experienced child psychiatrist.

### Measures

The main outcome was the number of days of hospitalization, following initial admission, used by the 12-month follow-up. The secondary outcome was the total number of hospital days used by the 12-month follow-up and the percentage of patients at full remission as defined by an EBW > 95% and an Eating Disorder Examination (EDE) global score within 1 standard deviation (s.d.) of expected norms (Cooper & Fairburn, 1987; Cooper *et al.* 1989; Fairburn & Beglin, 1994). EBW was calculated by expressing weight as a percentage of the expected weight corresponding to the 50th percentile for gender, age and height according to the CDC growth charts (Kuczmarski *et al.* 2000). This weight approximates the set point for return of menstruation in most females and is the weight where reversal in bone loss and growth resumption is likely to occur (Golden *et al.* 1997, 2008; Couturier & Lock, 2006; Le Grange *et al.* 2012*a*). The EDE is a structured and validated clinical interview assessing eating-related psychopathology and behaviors and is the standard outcome measure used in clinical trials of AN. An EDE score within 1 s.d. of community norms returns the risk of eating and weight concerns to community averages (Fairburn & Beglin, 1994).

The percentage of participants obtaining partial remission was also examined as defined by weight above 85% of EBW. This definition approximates the ‘intermediate outcome’ using Morgan–Russell criteria (Morgan & Russell, 1975) and is the DSM-IV weight cut-point for a diagnosis of AN (APA, 2000). It was included to allow comparisons with prior studies of adolescent AN. Other outcomes included percentage change in EDE global scores from baseline, readmission rates and the percentage of patients requiring treatment post-protocol.

### Diagnostic interviews

The child version of the EDE was used for participants aged ⩽14 years (Bryant-Waugh *et al.* 1996; Watkins *et al.* 2005) and the adult version (Fairburn & Beglin, 1994) for participants ⩾15 years. The Schedule for Affective Disorders and Schizophrenia for School-Aged Children (K-SADS-III) is a clinical interview for assessing psychiatric disorders in individuals aged 6–18 years; we used the Present and Lifetime version (K-SADS-PL; Kaufman *et al.* 1996). Both the patients and parents were interviewed at baseline with the composite ratings considered to ascertain co-morbid psychiatric diagnoses. Clinical assessment collected information including duration of illness, eating disorder symptoms, past medical history, and demographic and family information.

### Medical assessment

Height (±0.1 cm) was measured with a stadiometer (Holtain Ltd, UK). Body weight (±0.1 kg) was measured in light clothing using electronic scales (AND FW-150 K, Japan). Measurements were recorded at all five assessment points and before every FBT session. To assess medical stability, heart rate (beats/min) was reported as three times the recorded value for radial pulse measured for 20 s. Temperature was taken using a Becton and Dickinson electronic thermometer placed in the axilla. Blood pressure was recorded using a cuff covering two-thirds of the length of the right arm connected to a Space Lab Medical SL electronic sphygmomanometer.

### Psychometric questionnaires

To assess group differences and investigate change over time, an age-appropriate psychological questionnaire package was administered at each assessment point. Depression and anxiety symptomatology was assessed by the Revised Child Anxiety Depression Scale (RCADS; Chorpita *et al.* 2000, 2005), a 47-item, self-report questionnaire, designed for 7–18-year-olds to assess clinical syndromes based on DSM-IV criteria. Obsessive–compulsive disorder (OCD) symptoms were investigated using the revised Children's Obsessive Compulsive Inventory (ChOCI-R; Shafran *et al.* 2003; Uher *et al.* 2007), a self-report questionnaire that has good internal consistency and criterion validity. Self-esteem was assessed with the Rosenberg Self-Esteem Scale (RSES; Rosenberg, 1979), a measure commonly used in research and clinical practice.

### Treatments

#### Hospital care

Participants were admitted to one of two pediatric medical units under the care of specialist, multidisciplinary, eating disorder services. The two treatment centers have more than 30 years’ experience in managing adolescents with AN. Treatment is aimed at medically stabilizing patients and establishing safe eating. Both services are jointly led by an adolescent medicine physician and a child and adolescent psychiatrist. Patients are managed on a specialist program using a lenient behavioral approach. Patients admitted to the program attend a hospital-based school, a daily adolescent group program and a second daily physiotherapy program. Adolescent group activities include art and creative pursuits, psycho-education and psychological skills development. Patients are medically and psychiatrically reviewed on a daily basis with supportive psychotherapy provided by either a child and adolescent psychiatrist or a psychologist three times a week. All families were seen by a multi-disciplinary team for a comprehensive assessment during the admission and weekly family meetings were held with a focus on clinical update, psycho-education, nutritional education by an eating disorder dietician and preparation of families for out-patient FBT.

All patients were re-fed using a standardized protocol commencing with 24–72 h of continuous nasogastric feeds (ceased with daytime medical stability) followed by a combination of nocturnal nasogastric feeds and supported meals aiming for a total caloric intake of between 2400 and 3000 kcal/day. The amount and duration of nasogastric feeding was determined by markers of medical instability for a minimum of 14 days. Total caloric intake was based on a rate of weight gain of 1 kg/week (Kohn *et al.* 2011). Participants in the MS arm were subsequently discharged to out-patient FBT if they had no markers of medical instability for 72 h after nasogastric feeds were ceased. Participants in the WR arm continued in hospital on supported meals without nasogastric feeding once they had no markers of medical instability for 72 h, until they reached 90% EBW before discharge to out-patient FBT. Nasogastric feeding has been used routinely in medically unstable adolescents in pediatric treatment centers in Sydney, Australia and has been shown to be well tolerated by patients and their families, medically safe and lead to consistent early weight gain and MS (Halse *et al.* 2005; Kohn *et al.* 2011). Co-morbid psychiatric conditions were managed according to evidence-based practice including the use of psychotropic medications. Patients were readmitted if medically unstable or at acute psychiatric risk. Readmitted patients from both groups were treated identically, with medically unstable patients re-fed using nasogastric feeds and supported meals until medically stable, and psychiatrically unwell patients managed using evidence-based interventions with discharge based on treatment response and absence of thoughts of self-harm or suicide.

#### FBT

FBT is a three-phase treatment described in the *Treatment Manual for Anorexia Nervosa: A Family-based Approach* (Lock & Le Grange, 2013). In phase 1, parents are charged with taking responsibility for managing eating behaviors and weight gain. In phase 2, parents transition eating and weight control to the adolescent in an age-appropriate fashion. Phase 3 focuses on adolescent developmental issues. Treatment began within a week of hospital discharge and involved 20 sessions lasting 1 h each spread over a maximum of 12 months. The FBT protocol was considered complete if the family attended 20 sessions or if the goals of treatment were met prior to this, with treatment ended by mutual consent of the therapist, parents, medical team and investigators. If criteria for recovery were not met at session 20, families were offered further treatment, including FBT, and individual treatment for AN and co-morbid psychiatric conditions if appropriate.

FBT therapists were three psychologists and a social worker trained in the FBT model. The therapists attended a 2-day workshop, then spent 4 weeks administering FBT with an experienced therapist and had to meet clearly defined competencies (Rhodes *et al.* 2009) developed from the directives in the manual across each treatment phase (Lock & Le Grange, 2013) prior to treating randomized cases. Weekly individual and group supervision was provided by two experienced FBT therapists (A.W. and P.R.) with over 5 years of experience in FBT. Where consent was provided (89% of families), treatment sessions were recorded on digital video and a random sample of 5% of these sessions were assessed for treatment fidelity by one of the authors of the FBT manual (D.L.G.).

### Participant safety and re-hospitalization criteria

Participants in out-patient treatment were medically assessed on a weekly basis and if medically unstable (Katzman, 2005) or at acute psychiatric risk, were readmitted to hospital.

### Sample size and power

*A priori* power analysis estimated that a total of 36 patients per group completing the trial would provide 80% power to detect a group difference of 0.75 s.d. in the primary outcome (number of hospital days after initial discharge) (*α* = 0.05, two-tailed). Interim and end of recruitment analysis was conducted by a blinded data monitoring committee to guide recruitment closure.

### Statistical analysis

Baseline analysis used independent-samples *t* tests and one-way ANOVA for continuous variables and *χ*^2^ tests with Yates’ continuity correction for analysis of categorical data.

For the primary outcome (number of hospitalization days at the 12-month follow-up, after the initial admission) and for our secondary outcome (total number of hospitalization days at the 12-month follow-up), a linear regression analysis was used. In addition, we examined clinical outcome (full remission) using multivariate logistic regression. Full remission status at session 20, 6- and 12-month follow-ups was used as the multivariate binary outcome without imposing any longitudinal trend (time line varied considerably across treatment groups and across individuals). For partial remission the same approach was used. A linear regression analysis was used to compare weight change (%EBW) between groups at the end of hospitalization. A multivariate regression analysis was used to compare weight change between groups at session 20, 6- and 12-month follow-ups as the multivariate outcome. The same method was used to compare groups on the EDE global score at the three time points.

Potential moderators and mediators of treatment effects on full remission were examined by incorporating the MacArthur framework for moderator/mediator analysis (Kraemer *et al.* 2002, 2008) in multivariate logistic regression analysis of hospital utilization and full remission status at the three time points. Ten baseline variables were examined as potential intervention effect moderators: Site, %EBW, the ChOCI-R Obsession subscale, ChOCI-R Obsession Severity subscale, ChOCI-R Compulsion subscale, ChOCI-R Compulsion Severity subscale, RCADS Depression subscale, RCADS Anxiety subscale, RSES, and the EDE global scale). As these analyses were conducted as exploratory without particular *a priori* hypotheses, we used a nominal significance level (*α* = 0.05) and did not adjust for multiple candidate moderators. The baseline EDE global score was dichotomized at the median to depict the differential effect of treatment depending on the level of EDE global. The baseline ChOCI-R subscale score was dichotomized at the median to depict the differential effect of treatment depending on the level of compulsiveness. Both findings in moderator effects are presented in Table 3. For the mediator analysis, we examined one hypothesized mediator. In addition, the change in %EBW from baseline to end of hospitalization was examined as a potential intervention effect mediator.

### Ethical standards

All procedures contributing to this work comply with the ethical standards of the relevant national and institutional committees on human experimentation and with the Helsinki Declaration of 1975, as revised in 2008. This study was approved by the Human Research Ethics Committee of the Sydney Children's Hospitals Network, Westmead Campus, Westmead Hospital and the University of Sydney.

## Results

The only significant differences between study sites was the age of the participants, reflecting the different admission age criteria of each site (SCHN-W: mean = 14.14 years, s.d. = 1.07; WH: mean = 16.25 years, s.d. = 1.03, *t*_80_ = −8.644, *p* < 0.05, *η*^2^ = 0.48, very large effect) and the duration of illness prior to hospitalization, with patients from WH (mean = 9.83 months, s.d. = 8.29) diagnosed on average 3.4 months later than SCHN-W patients (mean = 6.42 months, s.d. = 4.24), (*t*_80_ = 2.07, *p* < 0.05, *η*^2^ = 0.05, moderate effect size). There were no differences in baseline variables (Table 1) or protocol adherence (Table 2) between treatment groups. Six patients (7.3%) were within 1 s.d. of community norms for the EDE at baseline; however, a comprehensive clinical diagnostic interview confirmed the diagnosis so these participants were included in the study.

**Table 1.** Demographic, clinical and psychological characteristics at baseline^a^^,^^b^

	SCHN-W		WH		Total		Total
	MS	WR	MS	WR	MS	WR	
*Demographics*							
Sample size	29	24	12	17	41	41	82
Age (years)^c^	14.36 (1.08)	13.87 (1.02)	16.16 (1.14)	16.30 (0.97)	14.89 (1.36)	14.88 (1.56)	14.89 (1.46)
Gender: male	1 (3.5)	0	1 (8.3)	2 (11.7)	2 (4.9)	2 (4.9)	4 (4.9)
Family structure (single parent/separated)	8 (27.6)	6 (25.0)	4 (33.3)	4 (23.5)	12 (29.3)	10 (24.4)	22 (26.8)
Ethnicity							
White	22 (75.9)	21 (87.5)	9 (75.0)	16 (94.1)	31 (75.6)	37 (90.0)	68 (82.9)
Asian	7 (24.1)	3 (12.5)	0	0	7 (17.1)	3 (7.3)	10 (12.2)
Other	0	0	3 (25.0)	1 (5.9)	3 (7.3)	1 (2.4)	4 (4.9)
Interpreter required during treatment	3 (10.3)	0	2 (16.7)	0	5 (12.2)	0	5 (6.1)
*Clinical characteristics*							
Co-morbidity							
Depression features	6 (20.7)	9 (37.5)	7 (58.3)	4 (23.5)	13 (31.7)	13 (31.7)	26 (31.7)
Self-harm/suicidality	8 (27.6)	9 (37.5)	6 (50.0)	6 (35.3)	14 (34.2)	15 (36.6)	29 (35.8)
Anxiety features	10 (34.5)	12 (50.0)	4 (33.3)	6 (35.3)	14 (34.2)	18 (43.9)	32 (39.0)
OCD	4 (13.8)	6 (25.0)	2 (16.7)	3 (17.7)	6 (14.6)	9 (22.0)	15 (18.3)
PTSD/trauma/grief	5 (17.2)	6 (25.0)	3 (25.0)	0	8 (19.5)	6 (14.6)	14 (17.1)
Developmental/intellectual concerns	0	2 (8.3)	2 (16.7)	3 (17.7)	2 (4.9)	5 (12.2)	7 (8.5)
Anorexia nervosa subtype							
Restricting only	20 (68.97)	17 (70.83)	9 (75.00)	11 (64.71)	29 (70.73)	28 (68.32)	57 (69.50)
Binge/purge	9 (31.03)	7 (29.17)	3 (25.00)	6 (35.29)	12 (29.27)	13 (31.73)	25 (30.51)
Excessive exercise	13 (44.83)	6 (25.00)	5 (41.67)	7 (41.18)	18 (43.90)	13 (31.71)	31 (37.80)
Duration of illness (months)^c^	6.72 (4.94)	6.04 (3.26)	9.00 (6.37)	10.41 (9.56)	7.39 (5.42)	7.85 (6.89)	7.62 (6.16)
Previous hospitalizations	3 (10.34)	1 (4.17)	0	1 (5.88)	3 (7.32)	2 (4.88)	5 (6.10)
%EBW	77.13 (6.61)	80.04 (5.34)	77.64 (7.07)	78.13 (6.72)	77.28 (6.67)	79.25 (5.95)	78.26 (6.35)
EDE global score^d^	2.88 (1.2)	3.06 (1.12)	3.14 (1.02)	3.36 (1.10)	2.95 (1.14)	3.19 (1.11)	3.07 (1.12)
EDE subscale: Restraint	3.44 (1.46)	3.71 (0.96)	4.00 (1.18)	3.93 (1.41)	3.60 (1.39)	3.80 (1.52)	3.70 (1.27)
EDE subscale: Eating concern	1.87 (1.29)	2.23 (1.22)	2.47 (0.68)	2.38 (1.02)	2.04 (1.17)	2.29 (1.13)	2.17 (1.15)
EDE subscale: Shape concern	3.33 (1.25)	3.37 (1.45)	3.07 (1.17)	3.59 (1.28)	3.25 (1.22)	3.46 (1.37)	3.36 (1.29)
EDE subscale: Weight concern	2.89 (1.48)	2.91 (1.54)	3.02 (1.77)	3.49 (1.38)	2.92 (1.55)	3.15 (1.49)	3.04 (1.51)
*Psychological characteristics*							
RCADS: Depression	56.21 (14.05)	57.13 (17.59)	62.75 (18.44)	56.35 (10.33)	58.12 (15.51)	56.80 (14.86)	57.46 (15.11)
RCADS: Anxiety	48.48 (11.98)	54.42 (16.89)	50.75 (14.02)	50.47 (9.46)	49.15 (12.47)	52.78 (14.28)	50.96 (13.45)
ChOCI-R: Frequency of obsessions	16.66 (4.17)	18.71 (7.30)	16.33 (2.61)	17.06 (4.42)	16.56 (3.75)	18.02 (6.26)	17.29 (5.18)
ChOCI-R: Severity of obsessions	5.34 (5.71)	7.58 (8.88)	5.42 (7.27)	6.65 (6.02)	5.37 (6.11)	7.20 (7.75)	6.28 (7.00)
ChOCI-R: Frequency of compulsions	23.66 (6.31)	27.25 (9.71)	27.25 (6.14)	26.59 (6.62)	24.71 (6.40)	26.98 (8.48)	25.84 (7.55)
ChOCI-R: Severity of compulsions	5.90 (6.76)	7.13 (7.12)	7.92 (8.04)	6.47 (4.61)	6.49 (7.11)	6.85 (6.15)	6.67 (6.61)
RSES	18.41 (6.67)	15.5 (7.12)	14.08 (6.57)	14.76 (6.18)	17.15 (6.85)	15.20 (6.68)	16.17 (6.80)

SCHN-W, Sydney Children's Hospitals Network, Westmead Campus; WH, Westmead Hospital; MS, medical stabilization; WR, weight restoration; OCD, obsessive–compulsive disorder; PTSD, post-traumatic stress disorder; %EBW, percentage expected body weight; EDE, Eating Disorder Examination; RCADS, Revised Child Anxiety Depression Scale; ChOCI-R, Children's Obsessive Compulsive Inventory Revised; RSES, Rosenberg Self-Esteem Scale.

a The *χ*^2^ test for independence was conducted to compare group differences for categorical variables and no statistically significant differences were found at the 0.05 level unless noted otherwise. Values given as *n* (%).

b An independent-samples *t* test was conducted to compare trial group mean differences for each continuous variable and no statistically significant differences were found at the *p* < 0.05 level unless noted otherwise. Values given as mean (s.d.).

c Statistically significant difference between study sites at the *p* < 0.05 level.

d Six patients (7.3%) were within 1 s.d. of community norms for the EDE at baseline.

**Table 2.** Treatment characteristics between groups at the 12-month follow-up

	MS		WR		Total	
	*n*	*n* (%)^a^ or mean (s.d.)^b^	*n*	*n* (%)^a^ or mean (s.d.)^b^	*n*	*n* (%)^a^ or mean (s.d.)^b^
Adherence to trial protocol	41		41		82	
Completed full protocol		36 (87.8)		33 (80.5)		69 (84.1)
Withdrew from FBT (<20 sessions)		4 (9.7)		5 (12.2)		9 (11.0)
Withdrew from in-patient care		1 (2.4)		3 (7.3)		4 (9.7)
Duration of first admission (days)^c^	40	21.73 (5.92)	38	36.89 (17.06)		29.12 (14.69)
Patients requiring readmission to 12-month FU	40	14 (35.0)	38	14 (36.8)		28 (35.9)
Readmission days to 12-month FU	40	22.78 (41.59)	38	27.51 (51.70)		25.15 (46.58)
%EBW change, admission to 12-month FU	40	17.77 (11.36)	38	15.75 (9.24)		16.78 (10.37)
EDE global score change, admission to 12-month FU	36	−1.53 (1.48)	33	−1.35 (1.58)		−1.44 (1.52)
FBT sessions^c^	36	24.25 (8.51)	33	31.30 (12.60)		27.62 (11.16)
Additional treatment required after FBT	36	11 (30.6)	33	15 (45.5)		26 (38.7)

MS, Medical stabilization; WR, weight restoration; FBT, family-based treatment; FU, follow-up; %EBW, percentage expected body weight; EDE, Eating Disorder Examination; s.d., standard deviation.

a The *χ*^2^ test for independence was conducted to compare group differences for categorical variables and no statistically significant differences were found at the 0.05 level unless noted otherwise.

b An independent-samples *t* test was conducted to compare trial group mean differences for each continuous variable and no statistically significant differences were found at the *p* < 0.05 level unless noted otherwise.

c Statistically significant difference at the *p* < 0.05 level.

**Fig. 2.**
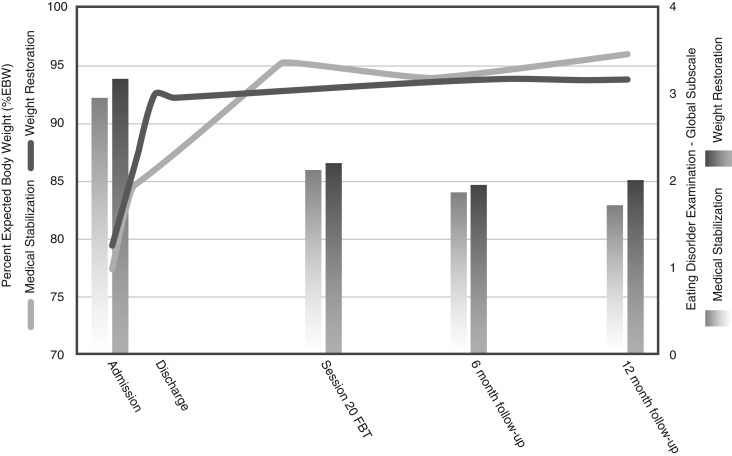
Remission indicators at each assessment point.

Table 3 and Figure 2 show the main analyses of the outcomes. Contrary to our hypothesis, there was no significant difference in hospital days used between groups following the initial admission (22.78 days for the MS group *versus* 27.51 days for the WR group; group difference = 4.74 days; *p* > 0.05, Cohen's *d* = 0.10, based on linear regression analysis). However, this resulted in significantly fewer total hospital days used at the end of the 12-month follow-up in the group randomized to MS (45.20 days for the MS group *versus* 65.50 days for the WR group; group difference = 20.20 days; *p* < 0.05, Cohen's *d* = 0.43, based on linear regression analysis). In addition, there was no significant difference in the rates of readmission between the two groups (36.1% in the MS group *versus* 33.3% in the WR group, *χ*^2^_1_ = 0.00, *p* = 1.00, n.s.). Of those readmitted, 70.8% were for weight loss and medical instability and 29.2% due to risk of self-harm or suicide. There was no difference between groups regarding the reason for readmission (*χ*^2^_1_ = 1.36, *p* = 0.18, n.s.).

**Table 3.** Estimated intervention effects (n = 82)

Outcome measure	MS	WR	Group difference	95% CI	Effect size^a^	*p* value
Number of hospital days						
12-month follow-up	45.20	65.50	20.2	0.3 to 40.1	0.43	**0.046**
Full remission %						
Session 20	25.00	21.20	−3.8	−23.7 to 16.1	26	0.709
6-month follow-up	22.50	28.20	5.7	−13.5 to 24.9	18	0.559
12-month follow-up	30.00	32.50	2.5	−17.8 to 22.8	40	0.809
Partial remission %						
Session 20	88.90	97.00	8.1	−3.7 to 19.9	12	0.180
6-month follow-up	82.50	87.20	4.7	−11.1 to 20.5	21	0.561
12-month follow-up	90.00	85.00	−5.0	−19.5 to 9.5	20	0.498
%EBW						
End of hospitalization	84.40	92.00	7.6	6.1 to 9.0	1.28	**<0.001**^b^
Session 20	95.20	93.10	−2.2	−5.8 to 1.5	0.27	0.240
6-month follow-up	93.90	93.70	−0.2	−4.4 to 4.0	0.02	0.923
12-month follow-up	95.50	93.60	−1.9	−6.1 to 2.4	0.19	0.390
EDE global score						
Session 20	2.12	2.22	0.10	−0.50 to 0.70	0.07	0.737
6-month follow-up	1.89	1.93	0.05	−0.45 to 0.54	0.04	0.857
12-month follow-up	1.73	2.01	0.28	−0.33 to 0.89	0.19	0.362

MS, Medical stabilization; WR, weight restoration; CI, confidence interval; %EBW, percentage expected body weight; EDE, Eating Disorder Examination.

Significant results appear in bold font.

a Effect size is the number needed to treat (NNT) for the binary outcomes (full and partial remission) and Cohen's *d* for the continuous outcomes (hospital days, %EBW, EDE global score).

b Difference planned by the trial arms of the study protocol.

More post-protocol sessions of FBT were used by the WR group (mean = 11.30 sessions *versus* mean = 4.25 sessions in the MS group; *t*_67_ = −2.75, *p* < 0.05, *η*^2^ = 0.10, large effect size). Other than the expected discharge protocol difference in %EBW (84.40% EBW in the MS group *versus* 92.00% EBW in the WR group) and associated length of admission (21.73 days in the MS group *versus* 36.89 days in the WR group) at the end of the initial hospitalization, there were no other significant differences between the groups on any outcomes at any time point.

Rates of full remission at 12 months post-treatment were not significantly different between the two groups (30.00% in the MS group *versus* 32.50% in the WR group). Rates of partial remission at 12 months post-treatment were also similar between the groups (90.00% in the MS group *versus* 85.00% in the WR group). An exploratory moderator analysis did not identify any moderators for the use of hospital days after initial discharge or total hospital days up to the 12-month follow-up.

An exploratory moderator analysis of full remission found that participants with greater baseline compulsiveness as measured with the ChOCI-R showed higher full remission under the MS condition compared to the WR condition at the 6-month follow-up (*p* = 0.036). This finding was consistent throughout the follow-up period, although the moderator effect was not statistically significant at session 20 (*p* = 0.262) or at the 12-month follow-up (*p* = 0.799). There were no statistically significant differences for moderating effects of symptoms of depression, anxiety or self-esteem on full remission.

## Conclusions

This study found that a longer initial hospitalization aimed at WR did not reduce the need for hospitalization, following the initial admission, over the course of care when adolescents with AN of less than 3 years duration were treated with FBT following hospitalization. The average number of hospital days used up to 12-month follow-up, following the initial admission, was 28 days in the WR condition and 23 days in the MS condition, with similar rates of readmission. As a result of the longer initial admission in the WR condition, the average number of hospital days used per participant at the 12-month follow-up was 65 days, 20 days more on average than those randomized to the MS condition. This difference in mean hospital days per participant was statistically significant, with a moderate effect size favoring the MS group.

As allowed in the protocol, additional FBT sessions were offered to families who had completed 20 sessions of manualized FBT without achieving remission from AN. Of note, participants in the WR group used more post-protocol sessions of FBT than those in the MS group. Why this difference emerges in unclear because those discharged at higher weights would be expected to need fewer sessions to achieve recovery. However, it is possible that parents whose children were discharged at higher weights did not perceive a need to act as definitively and expeditiously as those whose children were still underweight. Appropriate parental anxiety and concern is suggested as a key aspect of treatment engagement in the FBT manual (Lock & Le Grange, 2013). This area deserves greater investigation as these findings may assist in the optimization of FBT and additional cost savings to treatment. As there were no other differences in outcomes at follow-up, these results provide evidence that a brief initial hospitalization aimed at medical stabilization is effective when using FBT after discharge.

The difference in total hospitalization day use between the study arms has significant implications for the cost of treatment in AN. As out-patient care costs approximately 10% of the cost of in-patient care (Katzman *et al.* 2000), it is reasonable to use the difference in days of hospitalization as a proxy for the difference in costs for the treatment. In Australia, the daily State Price for the in-patient treatment of AN in adolescents is US$1252 (IHPA, 2014), representing an additional cost of US$25 000 for the WR group. Although variable, costs for pediatric eating disorder patients are considerably higher in the USA, with daily in-patient treatment costs between US$3590 (University Hospitals, 2014) and US$3979 for a non-monitored pediatric bed (Nationwide Children's, 2014). This would represent an additional cost of US$72 000 to US$80 000 per patient for the WR group within the first year of treatment. There are other potential negatives of prolonged admission, including reduced contact with family, friends, peers and educational facilities, with disruption of educational attainment, socialization and identity development (Meads *et al.* 2001).

Although not different between groups, our rates of full remission at the end of FBT, 6- and 12-month follow-ups were lower than in the one trial (Lock *et al.* 2010) that used the same definition (EBW > 95% and EDE within 1 s.d. of norm), which reported 12-month rates of full remission for FBT of 49.3% compared with 31.25% in the current trial. Rates of partial recovery (EBW > 85%) were similar (87.5% in the current trial *versus* 77.7% in Lock *et al.* 2010). In the other RCT of FBT to use a combination of weight and EDE scores (EBW > 95% and a global EDE score of within 2 s.d. of norm), a full remission rate of 67% was reported (Eisler *et al.* 1997). Using these criteria, 73.2% of participants in this trial would meet full remission criteria.

Using the standard of Lock *et al.* (2010), these poorer rates of full remission may be a result of higher levels of symptom severity in the current sample at randomization. Unlike other RCTs using FBT, all participants in this study were medically unstable on admission, an exclusion criterion in out-patient RCTs of FBT. Additionally, EDE scores were higher than those reported in the other similar trials (Eisler *et al.* 1997, 2007). These indicators of greater severity of illness in this study sample strengthen our findings, as this is the group most likely to require hospitalization during the course of illness.

There were no moderators or mediators of treatment effect identified for the use of hospitalization in the study. However, those with higher EDE global scores and higher reports of compulsive behaviors did better in the MS group. Although speculative, this moderator effect might be explained if longer hospitalization exacerbates and magnifies the rigidity and inflexibility common in AN (Le Grange *et al.* 2012*b*), as patients accommodate to in-patient routines.

The strengths of this study include the use of a randomized design, validated assessment and outcome measures, standardized and manualized treatment protocols, blinded assessments and multiple assessment points for follow-up. Additionally, treatment retention and outcome assessment rates were good.

There are limitations to this study that may affect the generalizability of the findings. The study was not designed as an equivalence trial and was only powered to detect a large difference between groups. It was conducted within an academic medical center that supported efficient referral to collocated FBT services, with FBT providers trained and supervised by experienced practitioners, a level of care that is not universally available. FBT is an empirically supported treatment and although other out-patient therapies might be useful, the relationship between other out-patient treatments and hospitalization was not examined.

The findings of this study are important for several reasons. This is the first study to apply a randomized protocol to different hospital interventions for adolescent AN. The results support shorter hospitalization for MS when out-patient FBT is available (Lock *et al.* 2008).

Controversy about the role of in-patient WR in the treatment of AN is long-standing (Silber *et al.* 1989; Crisp *et al.* 1991; Golden *et al.* 2003; Gowers *et al.* 2007), and although the current study cannot answer all questions, it provides important data suggesting that prolonged in-patient WR is not systematically beneficial in terms of clinical outcomes or cost-effectiveness for medically unstable adolescents with AN of less than 3 years’ duration who are receiving out-patient FBT. Although there is a clear need for medical hospitalization to treat medical instability in AN (Gowers *et al.* 2007), prolonged hospitalization is not only expensive but also seems to offer no treatment advantages when effective out-patient treatment is available. Previous studies suggest that psychiatric hospitalization in itself is not more effective than out-patient treatment for adolescent AN (Gowers *et al.* 2000), but none of these studies included medically unstable patients for whom hospitalization is considered essential. The current study describes an approach for the safe and efficient use of hospitalization for potentially life-threatening medical complications arising from extreme weight loss that could be implemented in treatment programs using FBT. Implementing treatment programs that integrate in-patient MS with out-patient FBT for adolescent AN of less than 3 years’ duration is likely to lead to more cost-effective care.
